# *Salmonella enterica* virulence databases and bioinformatic analysis tools development

**DOI:** 10.1038/s41598-024-74124-x

**Published:** 2024-10-24

**Authors:** Jing Han, Hailin Tang, Shaohua Zhao, Steven L. Foley

**Affiliations:** 1 Division of Microbiology, National Center for Toxicological Research, Food and Drug Administration, Jefferson, AR 72079 USA; 2 Division of Bioinformatics and Biostatistics, National Center for Toxicological Research, Food and Drug Administration, Jefferson, AR 72079 USA; 3 Office of Applied Science, Center for Veterinary Medicine, Food and Drug Administration, Laurel, MD 20708 USA; 4 Division of Microbiology, National Center of Toxicological Research, Food and Drug Administration, 3900 NCTR Rd, Jefferson, AR 7209 USA

**Keywords:** *Salmonella*, Virulence genes, Database, WGS analyses tools, Computational biology and bioinformatics, Microbiology

## Abstract

*Salmonella enterica*, a prominent foodborne pathogen, contributes significantly to global foodborne illnesses annually. This species exhibits significant genetic diversity, potentially impacting its infectivity, disease severity, and antimicrobial resistance. Whole genome sequencing (WGS) offers comprehensive genetic insights that can be utilized for virulence assessment. However, existing bioinformatic tools for studying *Salmonella* virulence have notable limitations. To address this gap, a *Salmonella* Virulence Database with a non-redundant, comprehensive list of putative virulence factors was constructed. Two bioinformatic analysis tools, Virulence Factor Profile Assessment and Virulence Factor Profile Comparison tools, were developed. The former provides data on similarity to the reference genes, e-value, and bite score, while the latter assesses the presence/absence of virulence genes in *Salmonella* isolates and facilitates comparison of virulence profiles across multiple sequences. To validate the database and associated bioinformatic tools, WGS data from 43,853 *Salmonella* isolates spanning 14 serovars was extracted from GenBank, and WGS data previously generated in our lab was used. Overall, the *Salmonella* Virulence database and our bioinformatic tools effectively facilitated virulence assessment, enhancing our understanding of virulence profiles among *Salmonella* isolates and serovars. The public availability of these resources will empower researchers to assess *Salmonella* virulence comprehensively, which could inform strategies for pathogen control and risk evaluations associated with human illnesses.

**Supplementary Information:**

The online version contains supplementary material available at 10.1038/s41598-024-74124-x.

## Introduction

As one of the major foodborne pathogens, *Salmonella enterica*causes about 1.35 million infections, leading to 26,500 hospitalizations and 420 deaths each year in the United States alone^1^. Currently, *S. enterica* encompasses more than 2,600 serovars that differ from each other in the polysaccharide portion of the lipopolysaccharide layer (O antigen) and/or the filamentous portion of the flagella (H antigen) present on the surface of *Salmonella*^2^. Different *S. enterica*serovars display variations in their pathogenicity, virulence, and susceptibility to antimicrobial treatment. While many serovars may be capable of causing infections in humans and animals, only a limited number of serovars cause most human infections^3^. Some *S. enterica*serovars are host-restricted and can only infect certain hosts, while others infect a wide range of hosts. Also, they vary considerably in the nature of the disease that they cause. Some serovars are more likely to cause invasive disease in humans, and some only cause mild gastroenteritis. These distinctions in disease severity may arise from the considerable genetic diversity observed in the virulence factors (VFs), which are molecules produced by microbial pathogens that allow them to overcome host defense mechanisms and cause disease in a host^4^. The difference in VFs among the serovars within *Salmonella*may impact their likelihood to cause more severe disease^5^.

Antimicrobial therapy is often required for the more severe cases of illnesses caused by *Salmonella*infection^6^. Therefore, the occurrence and spread of antimicrobial resistance (AMR) causes many concerns for making regulatory decisions related to antimicrobial use in food animals a continuing public health concern. Severe, often invasive, cases of *Salmonella*are typically associated with increased virulence of the infecting strain, which illustrates the important confluence of virulence and AMR^7,8^. Mechanisms for the spread of antimicrobial resistance characteristics among pathogens include both horizontal and vertical genetic transfer. Key components of horizontal gene transfer are plasmids, which are circular DNA elements that replicate independently from the chromosome and can acquire and exchange genes with the chromosome and other host-resident plasmids^9^. Like AMR genes, VFs can also be spread through horizontal and vertical genetic mechanisms to increase the likelihood that pathogens, such as *Salmonella*, develop an increased ability to colonize hosts and avoid immune system clearance while at the same time becoming resistant to antimicrobial treatment^10^.

Recent advancements in DNA sequencing have revolutionized our understanding of virulence and AMR in enteric pathogens, like *Salmonella*, offering profound implications for public health improvement. As DNA sequencing has become less expensive and more accessible, it has greatly accelerated biological and medical research and discovery. Whole genome sequencing (WGS), which can reveal the complete DNA make-up of an organism and facilitate the detection of variations both within and between species, has been widely used by public health and food regulatory agencies to expedite the detection and identification of bacteria isolated from food and/or environmental samples during foodborne disease outbreaks^11^. However, effective analyses of WGS data necessitates robust bioinformatics tools. Consequently, bioinformatics approaches are increasingly pivotal in exploring AMR and virulence in foodborne pathogens, including *Salmonella*. Despite the progress, existing tools for studying *Salmonella* virulence exhibit limitations in usability and result provision, hindering their efficacy in addressing public health inquires; thus, there is a pressing need to develop improved tools to enhance the assessment of potential virulence-associated factors. Several VF databases have been developed for enteric organisms, such as the Bacterial and Viral Bioinformatics Resource Center (BV-BRC; formally Pathosystems Resource Integration Center (PATRIC)), which curated *Salmonella* VFs based on WGS data, the Virulence Factor Database (VFDB), and Victors, which have curated large numbers of *Salmonella* VFs based on WGS data (over 4,000,000 *Salmonella*VF genes cataloged from over 5,000 genomes)^12,13^. While these sources offer vast amounts of data, the presence of redundant genes complicates navigation and use for virulence assessment. In addition, these databases lack comprehensiveness in some cases, as several VFs, including many that are encoded on plasmids, are not represented. To fully utilize the wealth of information provided by WGS data and accurately evaluate the virulence of *Salmonella* isolates, there was a need to develop an improved *Salmonella* database. Such a database should feature a non-redundant, comprehensive compilation of putative VFs from *Salmonella*, empowering more effective risk assessment.

In this study, a comprehensive *Salmonella* virulence database that contained virulence and putative VFs from both *Salmonella* genomic DNA and plasmids was developed. Additionally, computational tools that were devised for identifying virulence genes in *Salmonella* strains and comparing virulence gene profiles among different isolates were incorporated into the database. To validate the efficacy of the database and the tools, the WGS data from 43,853 *Salmonella* strains spanning 14 different key serovars were downloaded from NCBI and analyzed. The predicted virulence profiles formatted as binary data were further analyzed using BioNumerics (ver. 7.6; Applied Maths, Austin, TX) and Pandas (Python Data Analysis Library). Our results show that this newly developed database offers a valuable resource for the scientific community for rapid strain characterization and can assist in tracing the sources of pathogens based on their virulence profiles. The variations in VF profiles of different strains and serovars is also crucial for developing effective strategies for disease prevention, management, and treatment.

## Methods

### Generation of a non-redundant, comprehensive list of virulence factors from *Salmonella*

The generation of a non-redundant, comprehensive list of VFs from *Salmonella*employed two steps. In the first step, a list of putative virulence genes was generated based on the existing available datasets. Currently, there are several VF databases for enteric organisms, including PATRIC (now BV-BRC)^12^, VFDB^14^, and Victors^4^, that provided baseline virulence gene data for organisms such as *S. enterica*, *Escherichia coli*, *Shigella*, and other enteric pathogens. In the second step, additional *Salmonella*-associated VFs and other putative VFs, which are not represented in these databases, were identified through an extensive literature review^15^. In the initial phase of database development, *S.* Typhimurium LT2 was used as the reference genome for those VFs present in the strain. Additional genes, not in LT2, were subsequently added to the cumulative list, and their reference strain was provided in the database.

### Development of comprehensive *Salmonella* virulence gene databases

**Fig. 1:**
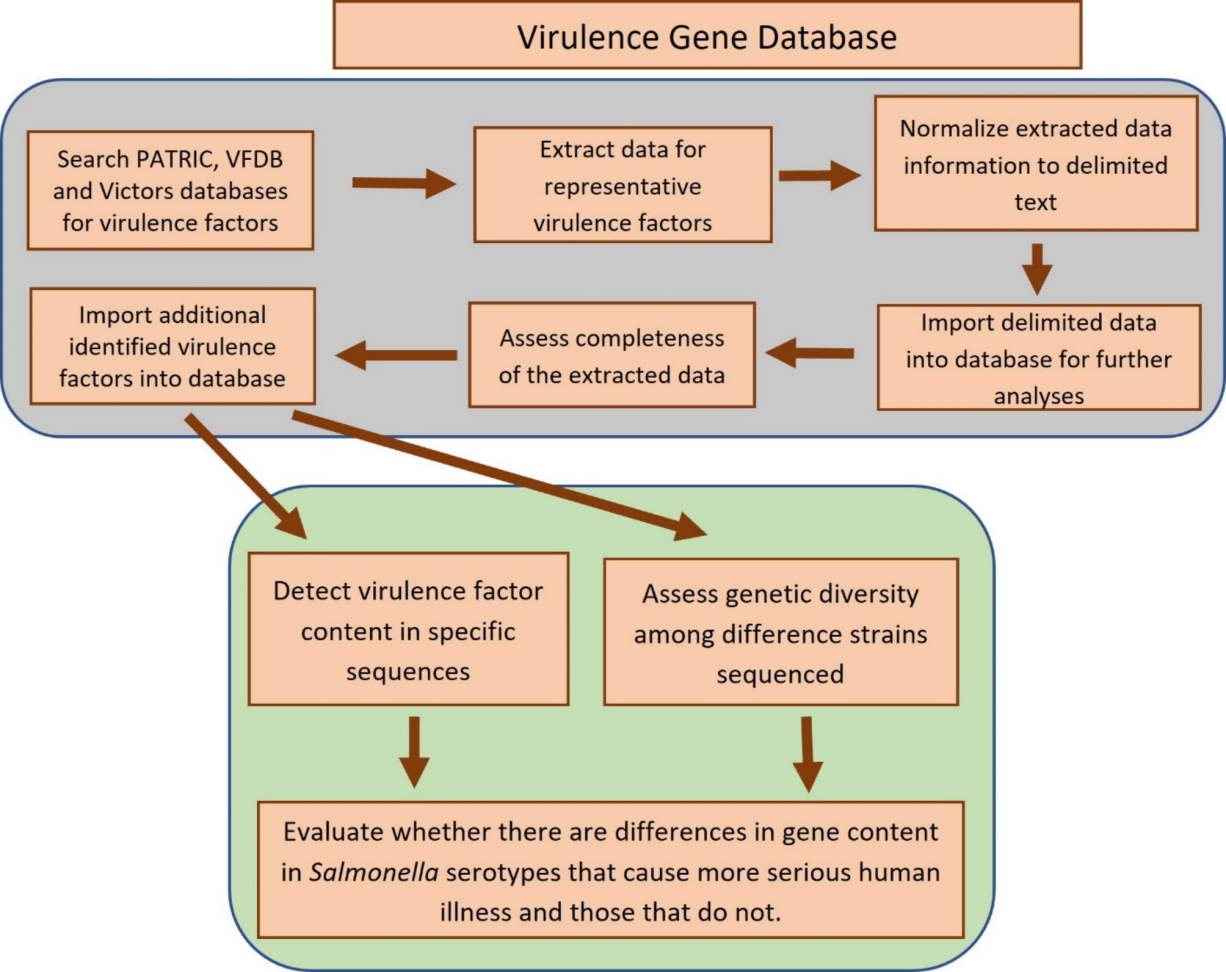
The scheme that was used to develop the virulence gene database and downstream application of the database.

The gene-level sequence data and other related information (protein sequence, product, etc.) of these genes were extracted from GenBank using a customized Python and Biopython program. The extracted data were normalized, a process of organizing the extracted DNA sequence data to ensure their consistency, reduce redundancy, and improve data integrity, then converted to a delimited form and imported into a PostgreSQL relational database. The completeness of the extracted data was regularly evaluated through ongoing literature review and the information on the additional identified virulence factors were subsequently imported into our database. The scheme used to develop the virulence gene database and downstream applications of the database are shown in Fig. 1.

### Bioinformatics analysis tools development

**Fig. 2:**
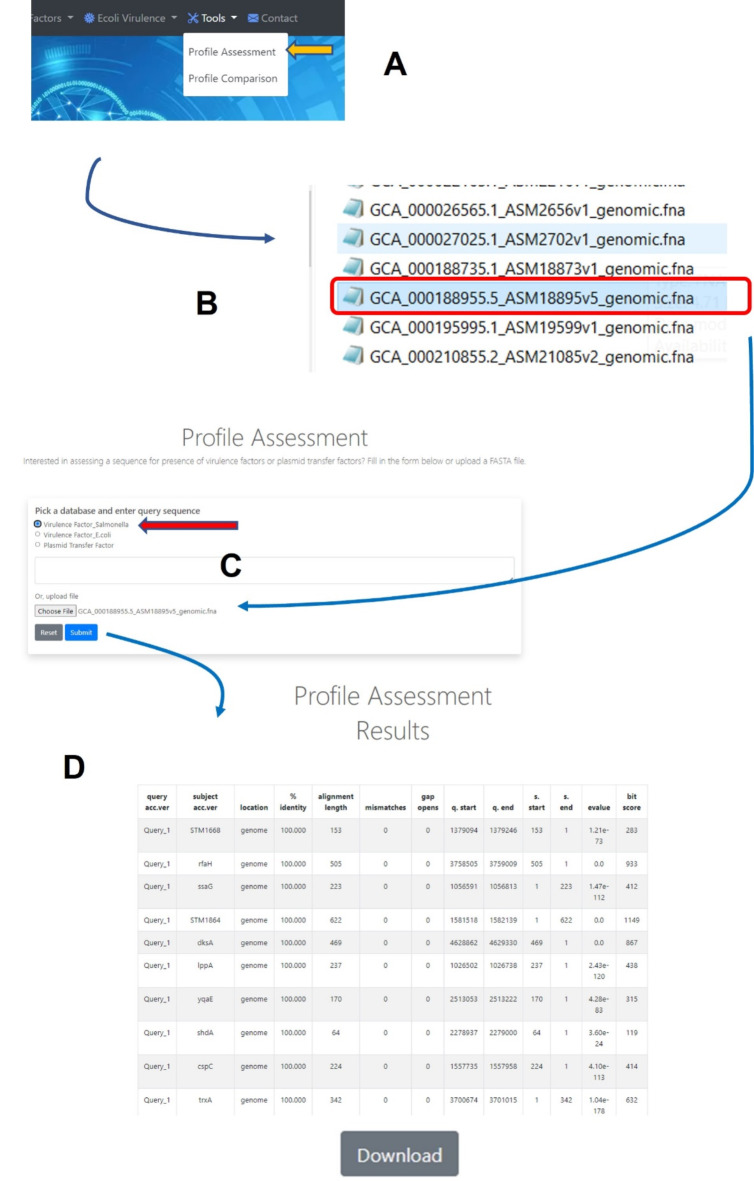
The analyses processes of the tool “*Salmonella* Virulence Factor Assessment” (**A-C**) and the output after the analysis (**D**). To start the analysis, “Profile Assessment” tab (**A**) is clicked, then a single sequence (FASTA) file of the isolate is selected (**B**) and “Virulence Factor_ Salmonella” (indicated by the red arrow) is picked. Then the sequence uploaded into system and a BLAST-based analyses is conducted. When a gene is identified information related to identity to reference, locations, etc. are provided for all the genes present (**D**). A download button is provided at the bottom of the output to facilitate further analyses in different programs.

Two distinct bioinformatics analysis tools were developed in this study. The first tool, termed “Profile Assessment” employs matching algorithms based on the BLAST algorithm to predict the presence of virulence genes in sequence strains. Users can copy/paste the sequence directly into a text box or input a single FASTA nucleotide file. Upon submission, the sequence is compared to the virulence gene sequences in the *Salmonella* VF database, and a detail table containing information on the identified virulence genes, including the % identity to the reference sequence, number of mismatched base pairs, e-value, and bit score, is generated (Fig. 2). A cutoff e-value of 10^−3^ is utilized, aligning with the threshold used in NCBI BLAST searching (https://biopython.org/docs/1.76/api/Bio.Blast.Applications.html). The genes with an e-value less than 10^−3^ indicate their predicted presence in the WGS and are included in the assessment result.

**Fig. 3:**
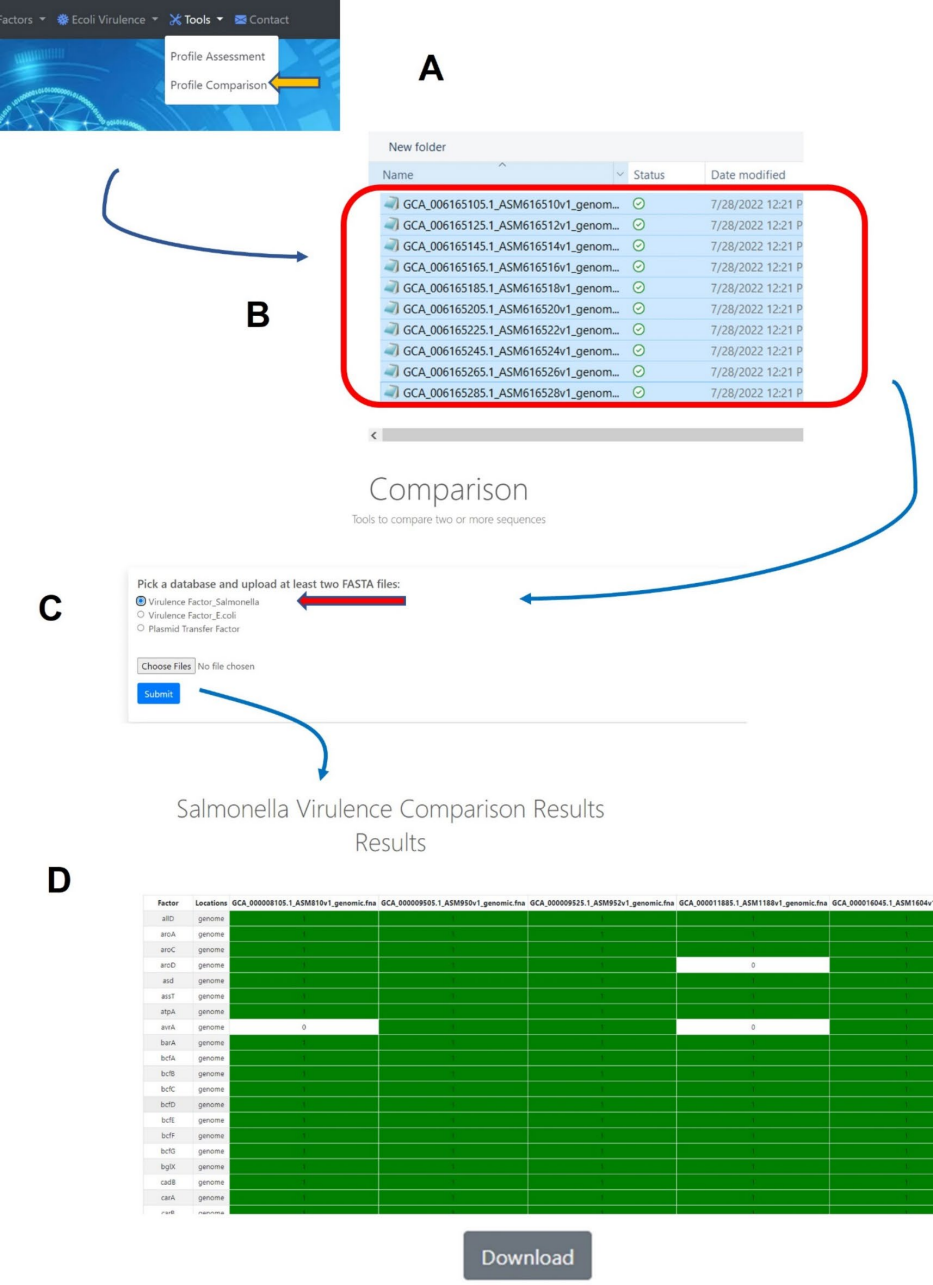
The analyses processes of the tool “Virulence Factor Comparison” (**A-C**) and the output after the analysis (**D**). To start the analyses, “Profile Comparison” tab (**A**) is clicked, then multiple sequence (FASTA) files of interest are selected for uploading (**B**) and “Virulence Factor_ Salmonella” (indicated by the red arrow) is selected to upload the sequences into system (**C**) and a BLAST-based analyses is conducted to identify the predicted genes present. When genes are detected a resulting presence/absence matrix is generated to facilitate comparison among strains (**D**). These analyses can be downloaded as a tab-delineated file using the download button and extracted for further phylogenetic analyses.

The second tool, named “Profile Comparison” was developed and can be used for comparative analyses of the virulence gene profiles among different strains. Users upload multiple FASTA-formatted sequence files simultaneously, which are then compared to the gene sequences in the VF database (Fig. 3). Similar to the VF Profile Assessment tool, genes are considered present if they meet the BLAST e-value cutoff of 10^−3^. The output of the comparison is a binary matrix indicating the presence or absence of the transfer genes in the sequences. The matrix can be viewed in the database window or downloaded from the database as a delimited file for further analysis.

### Assessment of the virulence database and analysis tools

To evaluate the virulence database and the functionality of the developed tools, WGS data from 43,853 *Salmonella* isolates from 14 different serovars (Table 1 and Supplemental Table 1), the most commonly identified serovars in human, animals and foods, that were released from 1980 (the earliest time of data available) until 2022 were downloaded from NCBI.

**Table 1:** Information on genomic sequences downloaded from NCBI for analyses of database and tools.

Taxon	Serotype	Number of Sequences	Assembly level*	Year released
58,095	Agona	3012	1,2,3,4	1980–2022
98,360	Dublin	2571	1,2,3,4	1980–2022
149,539	Enteritidis	692	2,3,4	1980–2022
149,385	Hadar	1505	1,2,3,4	1980–2022
611	Heidelberg	4502	1,2,3,4	1980–2022
440,524	I,4,[5],12:i:-	3889	1,2,3,4	1980–2022
286,783	Indiana	882	1,2,3,4	1980–2022
595	Infantis	11,821	1,2,3,4	1980–2022
363,569	Javiana	1595	1,2,3,4	1980–2022
108,619	Newport	6552	1,2,3,4	1980–2022
90,105	Saintpaul	2144	1,2,3,4	1980–2022
340,190	Schwarzengrund	2071	1,2,3,4	1980–2022
90,370	Typhi	1536	2,3,4	1980–2022
90,371	Typhimurium	1081	2,3,4	1980–2022
	Total:	43,853		

Notes: 1980 is the earliest date that sequences were available.

*Sequence assembly level: 1-contig; 2-scaffold; 3-chromosome; 4-completed.

For serovars of Typhimurium, Enteritidis, and Typhi, only the WGS of the sequence assembly levels 2–4 (i.e., scaffold, chromosome, and completed) were downloaded due to the large volume of available data, while all available sequence (levels 1–4, which also include contigs) were used for other serovars. For the evaluation of the *Salmonella* VF Profile Assessment tool, a random subset of WGS data from 810 strains was analyzed. The resultant percent identity to the reference gene sequences was extracted and merged with the results for the other strains for comparative analyses. For the *Salmonella*VF Comparison tool, the predicted virulence gene information was extracted from the database and transformed to binary data, with “1” indicating the presence of the gene and “0” indicating the absence of the gene. A random subset of 481 WGS were resampled and analyzed to confirm the detection of gene calls. The predicted virulence profiles (percent identity to reference for the assessment tool data or binary results for the comparison tool data) were further analyzed using BioNumerics (ver. 7.6; Applied Maths, Austin, TX) for Principal Component Analysis (PCA) and minimal spanning tree (MST) analyses to compare the similarity of the VF profiles within and among serovars. Additionally, Pandas (Python Data Analysis Library)^16^ was employed to identify the predominant VF profiles within each serovar, i.e., the genes that are present or absent in all the isolates of each serovar. From these results, one isolate representing the predominant VF profile in each serovar was chosen to create a dendrogram using BioNumerics to display the relatedness of their virulence profiles.

## Results

### Reference gene catalog composition, database construction, and analysis tools development

Based on the function of the genes from published literature^15,17–20^, a total of 594 VFs or putative VFs, including at least 150 located on a *Salmonella* Pathogenicity Island (SPI), 19 on *Salmonella* genomic island 1 (SGI) and at least 20 associated with plasmids from various *Salmonella*, were cataloged. The genes that encode these VFs were utilized to build the backbone of the *Salmonella* VF Database (Supplemental Table 2). As the field of *Salmonella* virulence research advances, ongoing efforts will be periodically assessed to evaluate and update the database with newly identified virulence genes. Figure 1 provides an overview of the workflow involved in this process. To effectively utilize the curated data from the initial phase of the study, the two distinct tools were developed to analyze WGS data. The *Salmonella* VF Profile Assessment tool is depicted in Fig. 2 and can be used to provide a detailed characterization of the virulence genes present in a sequenced strain. The *Salmonella* VF Profile Comparison tool (Fig. 3), facilitates simultaneous comparison of multiple FASTA files, generating a presence/absence matrix for comprehensive virulence gene profile comparisons among various *Salmonella* isolates.

To enhance user accessibility, a user-friendly website hosting the *Salmonella* VF gene data and analysis tools was built using Django v2.2 and running on Apache HTTP Server v2.4.37. The database and tools described above are publicly accessible at https://virulence.fda.gov and are freely available to the scientific community for identifying *Salmonella* VFs. Additionally, the database includes modules for assessing plasmid transfer genes and putative *Escherichia coli *VFs^21^. Notably, draft versions of the *Salmonella*virulence database have already contributed to published studies^22,23^.

### Evaluation of the *Salmonella* virulence factor assessment tool

**Fig. 4:**
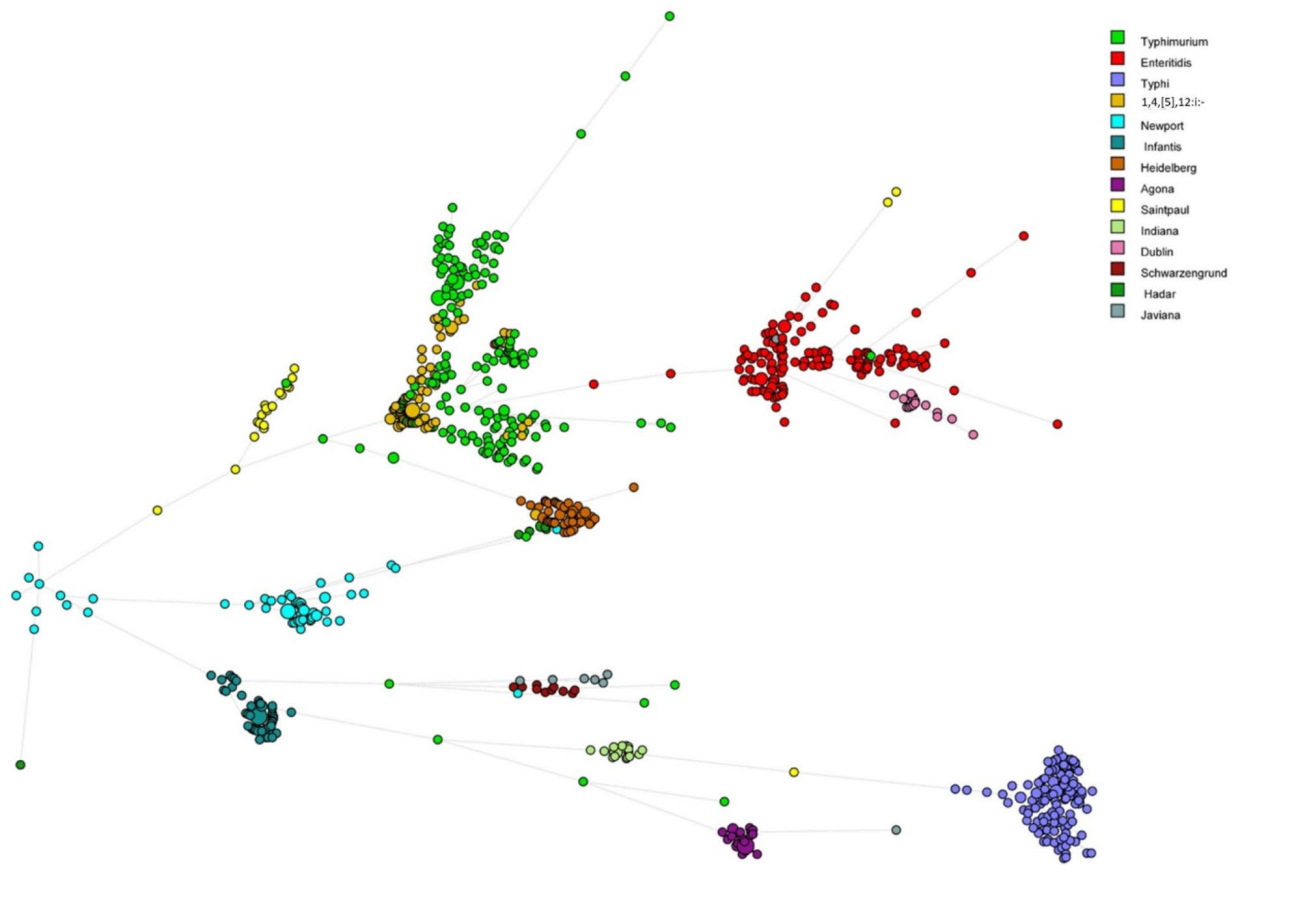
Minimal spanning tree of 810 strains randomly selected from one the 14 serovars. The minimal spanning tree was calculated based on the percent identity of the virulence factors to the references with the samples denoted by serovars.

The *Salmonella* VF Profile Assessment tool, featured in Fig. 2, provided a detailed assessment of the putative VF genes present in the analyzed FASTA file, delivering detailed information on the identified genes and their percent identity to the reference strains. The BLAST-generated output for the 810 strains analyzed, including the calculated percent identity for each detected gene, is shown in Supplemental Table 3. These data were compared across strains to assess the virulence gene similarity profiles among multiple *Salmonella* strains. The data imported into BioNumerics and MST were calculated based on the percent identity of the genes present compared to their references, with the samples denoted by serovar (Fig. 4). The results show that there is a general clustering based on serovars, with notable overlap between serovars Typhimurium and I,4,[5],12:i:-, which are known to be genetically closely related^24^. As the monophasic variant of *S*. Typhimurium, I,4,[5],12:i:- is closely related to *S*. Typhimurium both antigenically and genetically^25^. The key difference between them is that I,4,[5],12:i:- lacks the *fljB* gene, while the biphasic S. Typhimurium expresses two flagellar antigens: *fljC* (phase-1 flagellin) and *fljB*(phase-2 flagellin)^25^. In addition, serovars Enteritidis and Dublin exhibited close alignment and share similar antigenic profiles with one another.

### Evaluation of the *Salmonella* virulence factor comparison tool

The *Salmonella* VF Comparison tool, highlighted in Fig. 3, can generate a matrix indicating the presence or absence of genes in the database, which facilitates the simultaneous comparison of multiple FASTA files, enabling the comparison of VF profiles across diverse *Salmonella* strains from various serovars. Utilizing this tool, sequences from the 43,853 *Salmonella* strains (GenBank accession numbers provided in Supplemental Table 1) were analyzed to predict the presence/absence of the 594 VFs. The resulting binary data for the VF profiles was downloaded from the database and saved in an Excel format. When the subset of genes was resampled, the data matched the original calls. Subsequently, the presence rate of each gene in each serovar was calculated and summarized in Supplemental Table 4. Employing Pandas, the binary comparison data was further analyzed to identify the predominant virulence profile in each serovar. Table 2 presents number of the isolates with the predominant virulence profile along with their percentage within the serovar. The results revealed notable findings regarding the variations of VF profiles across different serovars. For instance, most of the *S*. Infantis isolates share the same VF profiles (86.47%, 9,637 out of 11,145) (Table 2). While in *S*. Newport and *S*. Saintpaul, the most common virulence profiles are only shared by 15.87% (1,039 out of 6,545) and 15.24% (327 out of 2,146) of the isolates, respectively (Table 2).

**Table 2:** The number of the isolates with the predominate virulence profile in each serotypes.

Serotype	Total # of isolates	Number of isolates with predominate virulence profile	Percent of total isolates	Representative isolate with the predominate virulence profile
Agona	3012	1729	57.40	GCA_007370785.1
Dublin	2571	1875	72.93	GCA_011286465.1
Enteritidis	692	323	46.68	GCF_002037995.1
Hadar	1505	1036	68.84	GCA_010737855.1
Heidelberg	4502	2955	65.64	GCA_007013425.1
I,4,[5],12:i:-	3889	1728	44.43	GCA_010399885.1
Indiana	882	662	75.06	GCA_024419575.1
Infantis	11,821	9637	81.52	GCA_006683165.1
Javiana	1595	474	29.72	GCA_010685105.1
Newport	6552	1039	15.86	GCA_011528265.1
Saintpaul	2144	327	15.25	GCA_010461045.1
Schwarz	2071	479	23.13	GCA_008642875.1
Typhi	1536	1173	76.37	GCA_003719235.1
Typhimurium	1081	301	27.84	GCA_001154285.1

**Fig. 5:**
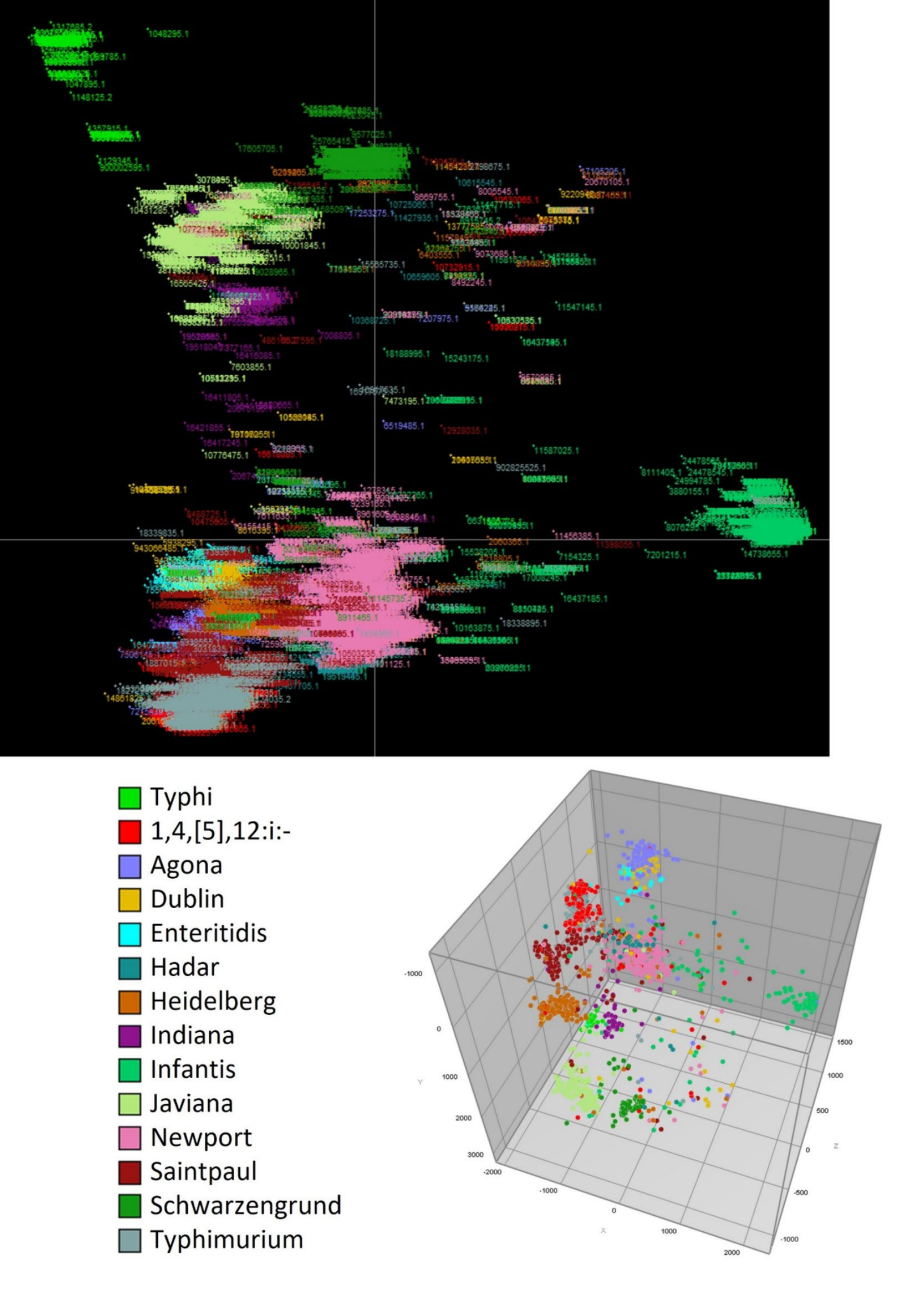
Principal component analysis (PCA) results of all the 43,853 isolates based on their VFs. The resultant PCA showed that the isolates of the same serovars are mostly clustered together, which indicates that the similarity of the VF profiles within the same serovars and diversity of the VF profiles among several of the different serovars.

**Fig. 6:**
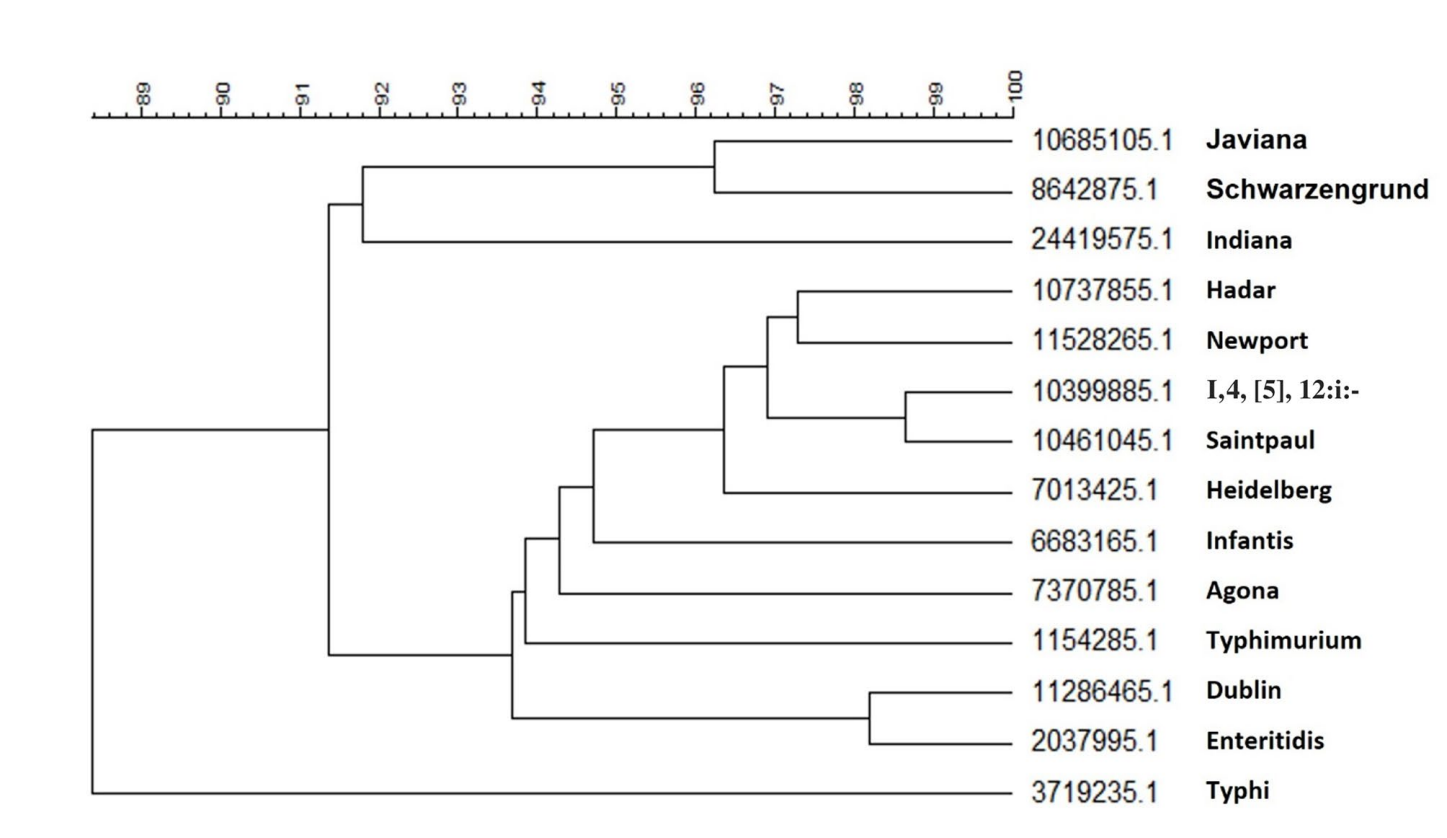
Phylogenetic tree of the predominate isolate from each serovar.

The data were further analyzed using BioNumerics with PCA. The resultant PCA showed that the isolates of the same serovars are mostly clustered together (Fig. 5). Based on their clinical presentation and host specificity, *Salmonella*is broadly categorized into two groups: typhoidal and non-typhoidal^15^. As the only typhoidal serovar analyzed, *S*. Typhi isolates are separated from the other nontyphoidal serovars (NTS). The typhoidal serovars also include Paratyphi A, B, or C that are most associated with causing typhoid or paratyphoid fevers. The NTS, including the other 13 serovars in this study, include the majority of the *Salmonella* serovars associated with foodborne infections. The result of the phylogenetic analysis of the most common virulence profile from each serovar shows that *S*. Typhi has less than 90% similarity with the isolates from the 13 NTS (Fig. 6). One of the main differences between *S*. Typhi and the NTS is the presence of the 28 genes (*stgABD*, STY_RS18755, *tviABCDE*,* vexABCDE*, and *pilLMNaNbOPQRSTUVV2*) in *S.*Typhi that are mainly associated with SPI-7, one of the SPIs that is particularly notable for its role in the virulence of typhoidal serovars^15,26^. These genes were detected in the majority (more than 97%) of the *S*. Typhi isolates examined. Conversely, the presence of these genes in the other 13 serovars is less than 5% (Supplemental Table 4) (Table 3).

**Table 3:**
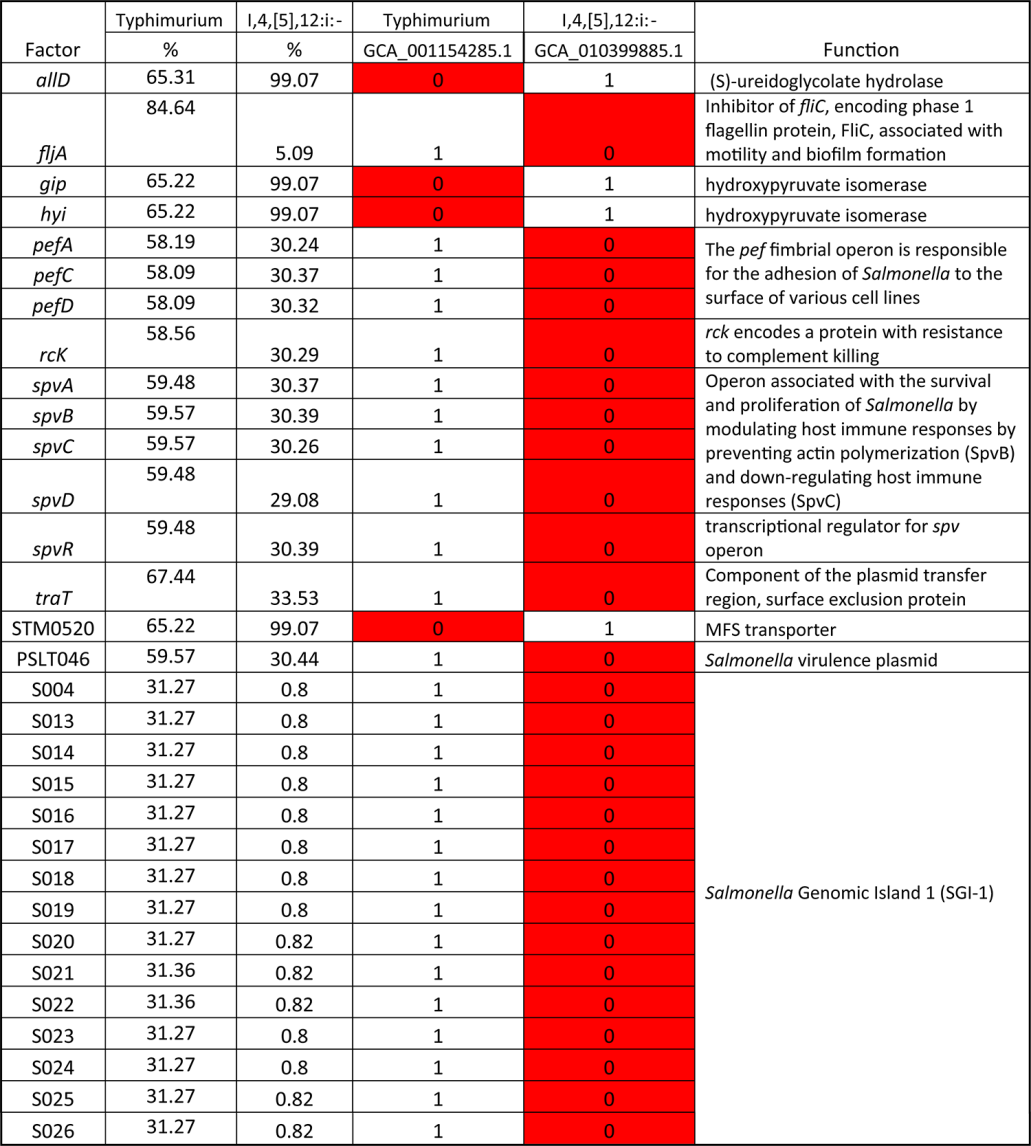
The genes with difference presence rate in serotypes Typhimurium and I,4,[5],12:i:-.

The PCA results showed that the isolates from *S.* I,4,[5],12:i:- overlapped extensively with the isolates from *S.* Typhimurium (Fig. 5), further confirming they are closely related. However, the phylogenetic analysis results showed the similarity between the predominant virulence profiles of these two serovars is only around 93.4% (Fig. 6). From the results of the phylogenetic analysis shown in Fig. 6, the predominant virulence profile of *S.* I,4,[5],12:i:- is also closely related to that of *S*. Saintpaul, with only 13 genes difference between their predominant VF genotypes (Table 4). Some key differences include the presence of the Stk fimbrial operon in *S.* Saintpaul and the *gogA* and *sseK* genes in *S.* I,4,[5],12:i:-. The VF profiles of isolates from serovars *S*. Enteritidis and *S*. Dublin are also similar based on the PCA and phylogenetic analysis, results with about 97.5% similarity (Fig. 6). The main differences are twelve genes, including plasmid encoded fimbriae (*pefACD)* and *rck* in *S.* Enteritidis and type VI secretion system (T6SS) genes (*sciRS* and *tssA*) common to *S.* Dublin, as shown in Table 5.

**Table 4:**
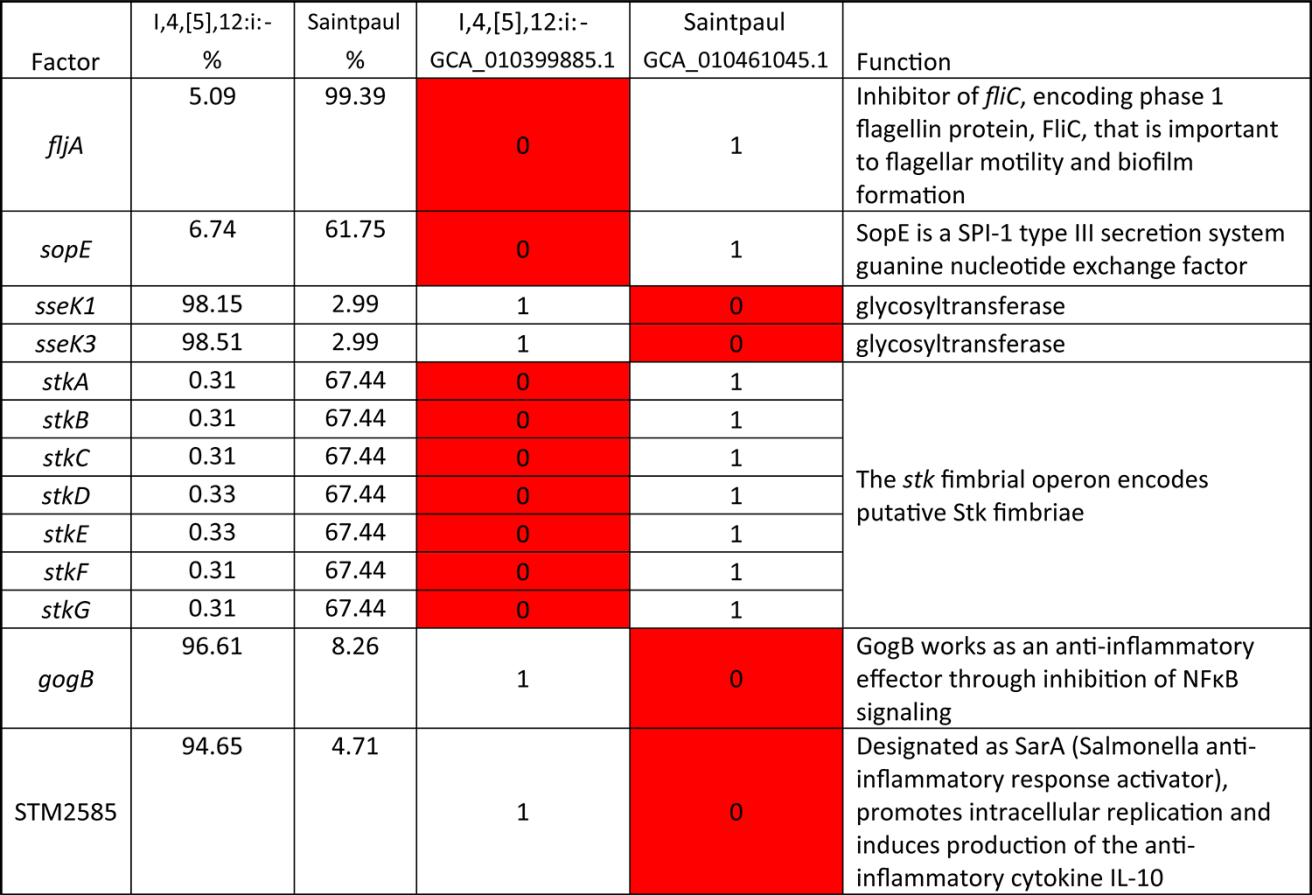
The genes with difference presence rate in serotypes I,4,[5],12:i:- and Saintpaul.

The binary comparison data of the comprehensive virulence gene profiles were also analyzed using Panda to identify the virulence genes that are present or absent in all the isolates of each serovar. The present analyses show that among the 594 virulence genes deposited in the VF database, 413 genes exist in all the isolates of at least one of the serovars. Nine of these genes (*pflB*, *narG*, *dsbB*, *slyD*, *citC*, *cpxR*, *focA*, *cspE*, and *cpxA*) were present in all the isolates analyzed, while 26 and 46 genes were found in all the isolates of 13 and 12 serovars, respectively (Supplemental Table 5). Additionally, there were 36 genes that were present in all the isolates of one particular serovar, but were detected much less frequently in other serovars. When looking at those genes absent in a particular serovar, there were 78 that were missing from all the isolates in at least one of the 14 serovars (Table 6). Isolates from *S*. Indiana were missing most of these genes, as it had 54 VFs absent from all the isolates analyzed from the serovar. All isolates of *S*. Schwarzengrund, *S*. Typhi, and *S*. Agona lacked 44, 38, and 33 of the VF genes, respectively. Conversely, *S*. Typhimurium and *S*. Infantis were the two serovars with the most virulence genes in any of the isolates, with only 12 and 13 genes missing, respectively.

**Table 5:**
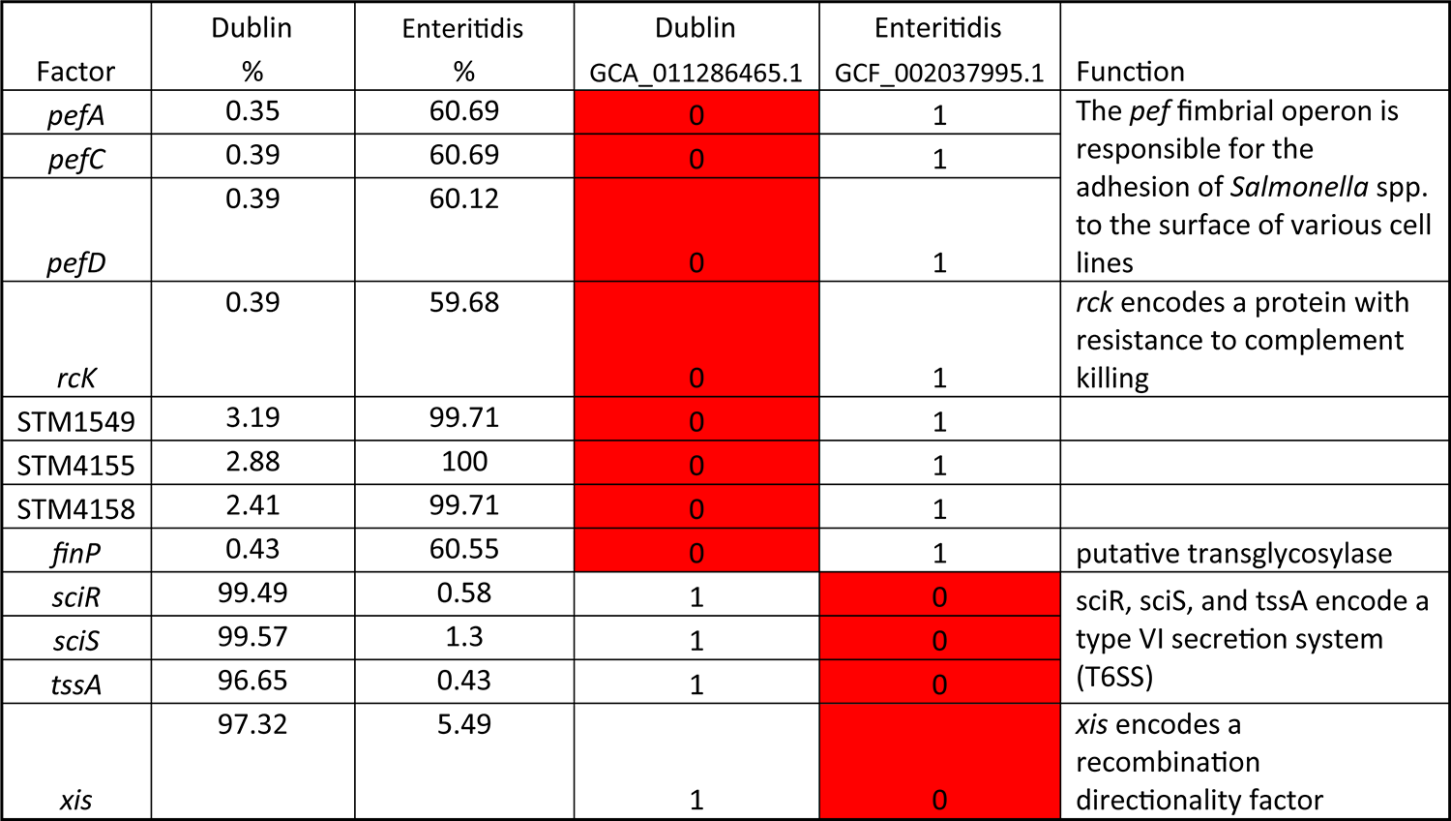
The genes with difference presence rate in serotypes Dublin and Enteritidis.

## Discussion

Not all the existing 2,600 *Salmonella* serovars exhibit equal pathogenicity to humans. Specific serovars or strains of *Salmonella*, especially those in subspecies enterica, are more apt to cause invasive infections in both humans and/or animals^27^. This feature suggests that these isolates may harbor specific VFs crucial for infection. In this project, WGS data from *S.* Typhi and 13 different NTS ranging from common causes of human illness (e.g. Enteritidis, Typhimurium and Newport) to thus less common (e.g. Schwarzengrund and Indiana) were analyzed using a database with a non-redundant, comprehensive list of *Salmonella* VFs and accompanying tools known as VF Profile Assessment and VF Profile Comparison tools. The database was created by compiling existing datasets and conducting an extensive literature review to account for those that were not represented in these databases. The current version of the database contains 594 VFs or putative VFs, including approximately 157 predicted to be located in an SPI-19 on SGI-1 and 21 that commonly located on plasmids (Supplemental Table 3). Some of these plasmid-associated genes, such as the *sit*operon, can also be located in the chromosome. Among the SPIs, genes from all 24 currently identified SPIs are included and more details about their functions was recently reviewed^15^. To establish the Virulence and Plasmid Transfer Factor Database to facilitate the prediction of virulence genes, the nucleotide and amino acid sequences of reference genes, and other related information such as the predicted product, locus tag, and accession numbers, were extracted from GenBank to create the backend reference VF dataset accessed by the analysis tools.

**Table 6:**
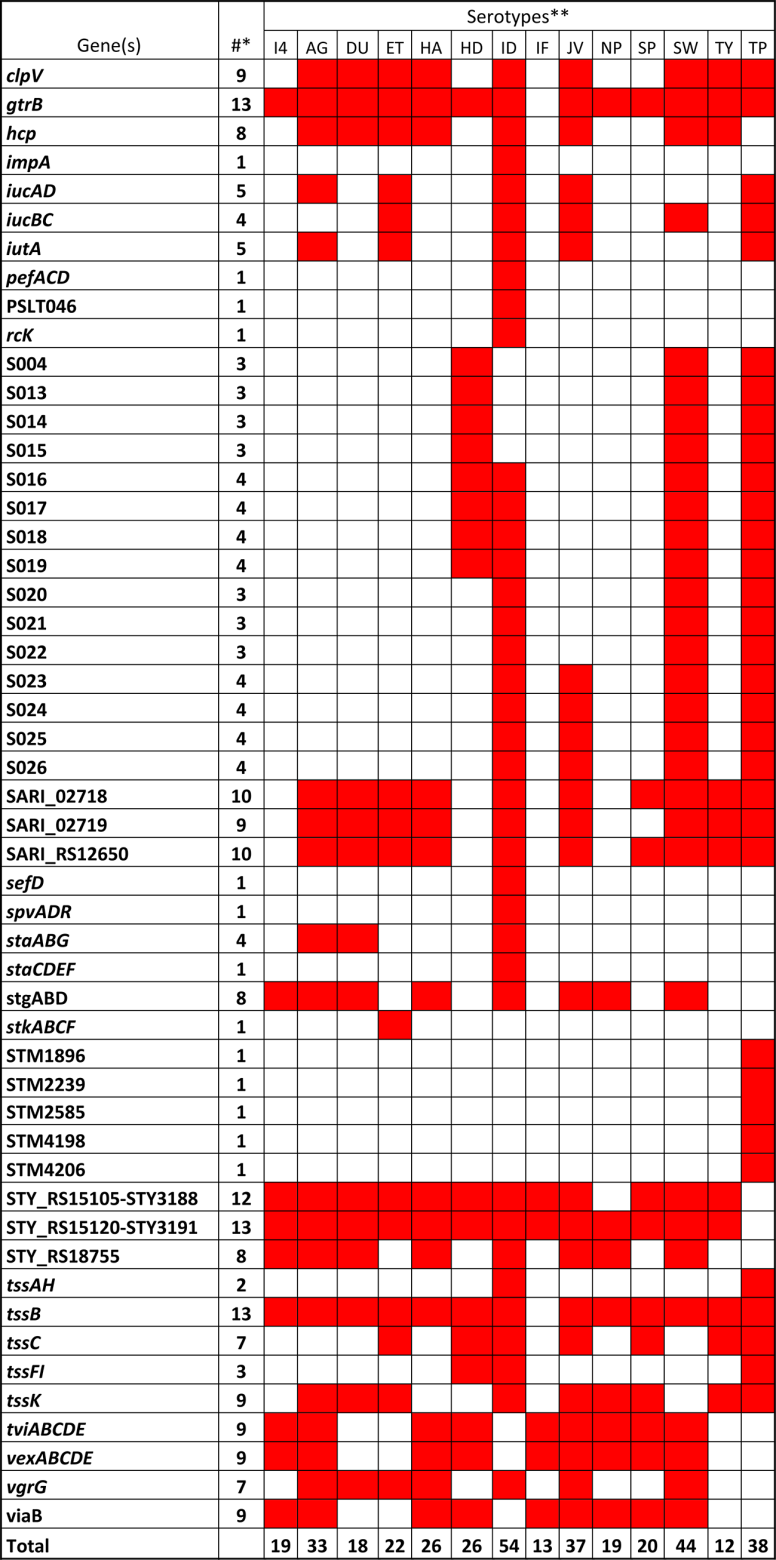
Genes that are missing in all the isolates analyzed in the serotypes. *The number of serotypes missing the genes. **Abbreviation for serotypes: I,4,[5],12:i:-; AG-Agona; DU-Dublin; ET-Dublin; HA-Hadar; HD-Heidelberg; ID-Indiana; IF-Infantis; JV-Javiana; NP-Newport; SP-Saintpaul; SW-Schwarzengrund; TY-Typhimurium; TP-Typhi. ***Genes that are missing are highlighted in red color.

The VF Profile Assessment tool was developed to facilitate the prediction of the presence of VFs in an uploaded sequence and provide detailed information on the nucleotide percent identity and matching location to the reference virulence genes in the database. The results of this tool can be viewed in the program online and/or downloaded and exported into a spreadsheet to facilitate further data analysis. To evaluate the utility of the tool, WGS data from 810 strains from 14 different serovars were combined and analyzed using Profile Assessment tool (Supplemental Table 3). An observed sequence diversity among individual virulence genes present in strains/serovars could offer valuable insights into their effects on host and/or tissue specificity, gene expression, and other related factors. For example, differences in the percent identities to reference genes among the various fimbrial gene (e.g., *bcf*operon, Supplemental Table 3) across different serovars may influence their tropism to interact with host epithelial cells^28^. The fimbriae can play key roles in adhesion to host tissues. With the capability of preferential binding to various glycans, fimbriae enhance *Salmonella’s*ability to adhere to different host cell surfaces, whether within the same host (tissue tropism) or across different hosts (host tropism), aiding in the colonization and infection process^28,29^. Figure 4 clearly showed that many VF similarity profiles within specific serovars cluster closely together while remaining distinct from those of other serovars. The pattern of serovar dispersion is similar to what is observed when examining the presence or absence of genes but reveals a greater diversity of genotypes. These data highlight that beyond simply identifying the presence or absence of particular VFs, understanding genetic diversity is crucial in shaping the pathogenicity and virulence of *Salmonella* strains.

The VF Profile Comparison tool allows users to upload multiple sequence files at once, which facilitated the comparison of multiple sequences simultaneously. The results display a binary matrix output indicating the presence or absence of VF genes across the uploaded sequences. The resultant comparison data can be visualized online in the program window or downloaded for further analyzed using other software programs. In this current assessment, WGS data of 43,853 *Salmonella* isolates from 14 different serovars were analyzed, extracted and collated in a spreadsheet and uploaded into BioNumerics for further analyses. The resulting PCA output demonstrated that isolates belonging to the same serovars predominantly clustered together (Fig. 5), suggesting a high degree of similarity in VF profiles within individual serovars and but diversity between the different serovars, with some exceptions noted above. Some of the key factors that drive the diversity among the serovars are the fimbrial operons present in the respective serovars (Supplemental Table 3). These phylogenetic results were consistent with the clustering generated by the Profile Assessment tool.

To assess the utility of the database for *Salmonella* characterization, differences in the VF profiles for the strains from the different serovars were compared in detail. Not surprisingly, the PCA results show that the *S*. Typhi isolates (*n* = 1, 536) separated from the NTS serovars (*n* = 42, 317). There were 28 genes present in the majority (more than 97%) of *S*. Typhi isolates but absent from the majority of other serovars that belong to three different gene clusters and their detailed functions are listed in Table 3. These included genes encoding a *S.*Typhi-specific fimbriae, type VI pilus, and the Vi antigen which are important for their pathogenesis^30–32^. Indeed, the Vi antigen production distinguishes *S*. Typhi from the NTS *Salmonella*^32^.

**Table 7:** The list of the genes that are in majority (more than 97%) of S. Typhi isolates, but less than 5% in other serotypes studied.

Genes	Functions
*stgA*,* stgB*, STY_RS18755, *stgD*	The *stg* gene cluster is S. Typhi -specific fimbrial operon. It expresses a functional adhesin that mediates adherence of serovar Typhi to host epithelial cells and inhibits phagocytosis, potentially contributing to the initial stages of typhoid fever pathogenesis
*pilV/pilV2*,* pilL*,* pilM*,* pilNa*,* pilNb*,* pilO*,* pilP*,* pilQ*,* pilR*,* pilS*,* pilT and pilU*	These genes belong to the *pil* operon, which encode proteins that are essential for the biogenesis of the R64 type IV pilus, which is used by S. Typhi to enter human intestinal epithelial cells.
*vexA*,* vexB*,* vexC*,* vexD*,* vexE*	encodes proteins required for transcriptional regulation of Vi production and biosynthesis (*tviABCDE* genes) and translocation (*vexABCDE* genes).
*tviA*,* tviB*,* tviC*,* tviD*,* tviE*	

The differences in the VF profiles of the representative isolates with the predominant virulence profiles from *S. Typhimurium* and *S.* I,4,[5],12:i:- were analyzed, and the genes unique to each isolate are listed in Table 3, along with their overall prevalence in these two serovars. Although there are 31 virulence genes listed as different between the predominant virulence profiles of these two serovars; with the exception of *fljA*, the overall presence rates of the other genes are not significantly different between the serovars. This further confirmed that the monophasic variant of *S.* Typhimurium, *S.* I,4,[5],12:i:- is closely related to *S.* Typhimurium. While four genes (*allD*, *gip*, *hyi*, and STM0520) are absent in the predominant virulence profile of *S*. Typhimurium, their presence rates in all the *S.* Typhimurium isolates analyzed in this study are more than 65%. Meanwhile, the other 27 VFs are absent in the predominant profile of *S.* I,4[5],12:i:- and present in the predominant virulence profile of *S.* Typhimurium, but their presence rates in all the *S.* Typhimurium isolates analyzed are relatively low. Except for *fljA*, the presence rates of each of the other 26 genes in the *S.* Typhimurium isolates is less than 60% (Table 3). The reasons for this phenomenon are that the VF profiles of *S*. Typhimurium are notably diverse, with a total 227 distinct VF profiles identified among the isolates analyzed (*n* = 1,081), and the predominant profile of *S.* Typhimurium are only present in 27.94% (302/1,081) of the strains. Notably, the majority of the 27 genes that are absent in the predominant VF profile of *S.* I,4,[5],12:i:- are located on pSLT virulence plasmid or SGI1. The *spv* locus (genes *spvABCD* and *spvR*), which is strongly associated with strains that cause NTS bacteremia and not present in typhoid strains, is missing in the majority (around 70%) of *S.* I,4,[5],12:i:- and 40% of *S.* Typhimurium isolates. The *spv* operon is associated with the survival and proliferation of *Salmonella spp*. within macrophages^33^. It encodes the primary virulence factors associated with serovar-specific virulence plasmids in *S. enterica*. The loss of the *spv* region eliminates the virulence phenotype of the serovars in their animal hosts and frequently in the mouse model, introducing a pSV (*Salmonella*virulence plasmid) into a serovar naturally lacking it does not enhance the virulence properties of the strain, which implies that other chromosomally encoded factors are essential for the virulence phenotype^19^. The low presence rate of the *spv* locus in *Salmonella* shown in this study is consistent with earlier research indicating that only a small fraction of *Salmonella*serovars contain this virulence operon^34,35^. Another operon that is missing from the predominant virulence profile of *S.* I,4,[5],12:i:- and has a low presence rate in both serovars is the *pef* fimbrial operon (*p*lasmid-*e*ncoded *f*imbriae), which is responsible for the adhesion of *Salmonella*spp. to the surface of various cell lines^15,36^. Since most plasmids impose fitness costs on their hosts, the loss of the plasmid-encoded VFs in *Salmonella* isolates may have evolutionary advantages that have resulted in its emergence over the past decade. Also, the genes located on SGI-1, a genomic island containing an antibiotic resistance gene cluster, are missing in 99% of the *S.* I,4,[5],12:i:- isolates and exist in only around 31% of *S.* Typhimurium isolates. Other VFs that have lower presence rates in *S.* I,4,[5],12:i:- include *fliA*, *rck*, and *traT*. *fljA* encodes an inhibitor of *fliC*, which encodes a phase 1 flagellin protein, FliC, that is important to flagellar motility and biofilm formation^37^. This result is consistent with the previous finding that *S.* I,4,[5],12:i:- is closely related to *S*. Typhimurium but lacks the expression of *fliA* and *fljB*(encoding phase 2 flagellin) common to all Typhimurium isolates^24^. *rck* is located close to the *pef*operon on pSV, and it encodes a protein with resistance to complement killing that can recruit various complement inhibitors to resist the attack of the innate immune system and has been implicated in the invasion of epithelial cells^15^. *traT*encodes a 27 kDa protein that imparts weak resistance to serum killing and is a component of the plasmid transfer region^15^.

The major difference between the VF profiles of *S.* I,4[5],12:i:- and *S*. Saintpaul is the presence of a fimbrial gene cluster (*stkABCDEFG*) that occurs in about 67% of *S*. Saintpaul isolates, but only about 3% in *S.* I,4[5],12:i:- (Supplemental Table 4). The *stk* fimbrial operon encodes putative Stk fimbriae and was initially reported to be specific for *S*. Paratyphi A^38^. However, subsequent studies revealed the presence of this operon in other NTS, such as *S*. Heidelberg, and *S*. Kentucky^38^. Our results showed that this operon has high presence rates in serovars Hadar, Indiana, and Heidelberg with a presence rate of more than 99% in *S*. Hadar and *S*. Indiana and more than 97% in *S*. Heidelberg (Supplemental Table 4). The presence rate in the rest of the serovars analyzed in this study was around or less than 1%. The genes that are missing in almost all *S*. Saintpaul isolates, but present the great majority of *S.* I,4[5],12:i:- are the T3SS effectors *sseK1* and *sseK3.*These effectors encode SseK proteins, which are reported to help inhibit antibacterial and inflammatory host responses^15,39^. While the presence rate of *sseK1* and *sseK3* in *S*. Saintpaul is only about 3%, another gene variant, *sseK2*, is detected in more than 99% of all serovars analyzed in this study, including *S*. Saintpaul (Supplemental Table 4). In all the isolates analyzed in this study, the presence rate of *sseK1* and *sseK3* is consistent, either more than 99% or less than 3% in a particular serovar. This phenomenon is logical given the collaborative inhibition of the NF-κB signaling pathway by SseK1 and SseK3 during *Salmonella*infection in macrophages^19^. Although SseK2 can inhibit TNF-α-induced NF-κB reporter activation, its impact on the NF-κB pathway during *Salmonella*infection in macrophages is minimal^15^. Further research is needed to explore the role SseK2 plays in *Salmonella* virulence, considering its high prevalence in *S. enterica* strains. The other two genes, *gogB*and STM2585, encode T3SS effectors that are involved in the inflammatory response^40^.

The differences in the major VF profiles between serovars *S*. Enteritidis and *S*. Dublin are due to 12 genes (Table 5). The plasmid encoded fimbriae genes (*pefACD*), and resistance to complement killing (*rck*) are missing from more than 99% of the *S*. Dublin isolates. Conversely, the genes *sciR*, *sciS*, *tssA*, and *xis* are missing from the majority isolate of *S*. Enteritidis, but present in greater than 97% of *S.* Dublin. The genes *sciRS*, and *tssA* encode a T6SS, which is a contact-dependent contractile apparatus that contributes to *Salmonella*competition with the host microbiota and its interaction with infected host cells^41–44^. These findings highlight that these *S*. Enteritidis lack the T6SS, which is consistent with the previous finding that several genomic islands appear absent or degenerate in *S.*Enteritidis^45^.

*Salmonella* virulence systems are very complex, as many genes are involved in contributing to their virulence. Numerous VFs, including adhesion molecules, invasins, lipopolysaccharides, polysaccharide capsules, iron acquisition factors, host defense-subverting mechanisms, and toxins, have been identified in *Salmonella*, and these VFs play different roles during infection to enable the bacterial cells to colonize the host, disseminate, and cause disease^15^. The difference in the presence/absence of the virulence genes in each isolate/serovar might indicate their relative virulence to humans or other animal species. Therefore, the development of enhanced tools to identify *Salmonella* VFs can help to predict virulence potential and explain the observed clinical disparities in disease pathogenesis, which is important to understand risks associated with different *Salmonella* genotypes.

## Conclusions

*Salmonella enterica* remains a significant contributor to foodborne illnesses worldwide, necessitating a deeper understanding of its virulence potential. These insights are crucial for developing targeted prevention and treatment strategies, improving vaccine design, and enhancing public health interventions to better control and reduce *Salmonella*-related infections. By comprehensively analyzing virulence gene profiles, researchers can identify mechanisms that contribute to the ability of *S. enterica* to cause disease and better assess the potential risk of causing disease in humans posed by the different *Salmonella* serovars as well as through specific *S. enterica* strains. The *Salmonella* Virulence Database and tools evaluated in the current project offer invaluable bioinformatic resources for identifying and characterizing these virulence genes. Making these resources publicly accessible empowers FDA scientists and the wider scientific community to comprehensively evaluate *Salmonella* virulence. Moreover, the tools’ capacity for phylogenetic comparison of virulence genes across various strains and serovars, particularly between isolates that cause severe disease and those associated with milder illness, will facilitates the identification of key VFs that influence and contribute to infection and disease severity. These attributes will enhance our capacity to effectively mitigate *Salmonella*-related health risks.

While this study significantly advances our understanding of the virulence of *S. enterica* isolates, there are limitations that require further investigation. For instance, although the current database includes a comprehensive list of virulence factors, it largely depends on existing information and may miss novel virulence factors that have yet to be identified or characterized, particularly in emerging or less-studied *Salmonella* serovars. Ongoing literature review and mining of WGS data from emergent pathogens will be essential to capture these newly discovered VFs. Additionally, although the bioinformatics tools are effective in identifying virulence genes, their presence does not guarantee that they are expressed or functional. Future research could address these limitations by incorporating experimental approaches, such as in vitro and in vivo assays, to validate bioinformatics predictions and uncover new virulence mechanisms.

## Electronic supplementary material

Below is the link to the electronic supplementary material.

Supplementary Material 1

Supplementary Material 2

## Data Availability

All data is publicly available through GenBank and identified in the Supplemental Tables of the manuscript. The DNA and amino acid sequences of the database genes are provided in Supplemental Tables 2 and at the database website (https://virulence.fda.gov). Supplemental Table 2 also provides the GenBank accession number and locations of the reference genes and corresponding protein IDs for the virulence factors included in the database. Supplemental Tables 1 and 3 includes the GenBank Assembly Accession numbers for the genomes used for the evaluation of the Virulence Factor Profile Comparison and Virulence Factor Profile Assessment tools, respectively.
