# Supplementary material for: Salmonella enterica virulence databases and bioinformatic analysis tools development
Source: Sci Rep. 2024 Oct 24;14:25228. doi: 10.1038/s41598-024-74124-x (PMC11502889; doi:10.1038/s41598-024-74124-x)
Supplement: Supplementary file 2 — Supplementary Material 2 [file 41598_2024_74124_MOESM2_ESM.docx]

**Supplementary Materials:**

Supplemental Table 1. Strains used in the study to evaluate the *Salmonella* Virulence Factor Database.

Supplemental Table 2. Virulence or putative virulence genes in the *Salmonella* database.

Supplemental Table 3. Percent nucleotide similarity to reference genes for a subset of isolates from different serotypes.

Supplement Table 4. The presence rates of each virulence genes in each serotype.

Supplemental Table 5. The genes that exist in all the isolates of this serotype.
